# Edible Insects: Global Research Trends, Biosafety Challenges, and Market Insights in the Mexican Context

**DOI:** 10.3390/foods14040663

**Published:** 2025-02-15

**Authors:** Keyla Cruz-García, Yolanda Donají Ortiz-Hernández, Marco Aurelio Acevedo-Ortiz, Teodulfo Aquino-Bolaños, Tlacaelel Aquino-López, Gema Lugo-Espinosa, Fernando Elí Ortiz-Hernández

**Affiliations:** 1 Instituto Politécnico Nacional, CIIDIR Unidad Oaxaca, Santa Cruz Xoxocotlán 71230, Oaxaca, Mexico; kcruzg2002@alumno.ipn.mx (K.C.-G.); taquino@ipn.mx (T.A.-B.); taquinol1800@alumno.ipn.mx (T.A.-L.); 2 Secretaría de Ciencias, Humanidades, Tecnología e Innovación (SECIHTI), Instituto Politécnico Nacional, CIIDIR Unidad Oaxaca, Santa Cruz Xoxocotlán 71230, Oaxaca, Mexico; glugoe@ipn.mx; 3 Instituto Politécnico Nacional, ESIME Culhuacán, Coyoacán 04440, Ciudad de México, Mexico; fortizh@ipn.mx

**Keywords:** edible insects, entomophagy, bibliometric analysis, food security, global trends, sustainable protein, biosafety

## Abstract

The growing global interest in edible insects as a sustainable protein source has positioned them as a promising solution to food security challenges. In Mexico, entomophagy is deeply embedded in cultural traditions, particularly in Oaxaca, where grasshoppers, leafcutter ants, and red agave worms form an integral part of the region’s intangible heritage. This study conducted a bibliometric analysis of global research on edible insects (2009–2023) using Scopus and tools such as VOSviewer and Bibliometrix to analyze 218 publications. The analysis highlighted research trends, influential authors, and key themes, including nutrition, biosafety, and sustainability. To complement the bibliometric study, an exploratory analysis of edible insect commercialization in Oaxaca was conducted, focusing on virtual platforms and local markets. The findings reveal consistent global growth in edible insect research, with Mexico contributing six publications between 2020 and 2023. Despite advancements in safety standards and regulatory frameworks globally, Mexico still lacks formal sanitary controls and regulations for insect-based products. Nevertheless, its diverse commercialization efforts and rich cultural heritage, particularly in Oaxaca, showcase its potential to bridge tradition and innovation. This study highlights the urgent need for regulatory frameworks and research capacity to ensure safety, preserve cultural identity, and sustainably expand Mexico’s edible insect market.

## 1. Introduction

The rising global demand for sustainable protein sources, driven by rapid population growth and environmental degradation, has positioned edible insects as a promising alternative to conventional animal proteins [1,2]. The Food and Agriculture Organization (FAO) projects that by 2050, the global population will exceed 9 billion people [1,3]. This population surge is expected to create significant food security challenges, particularly in low-income countries, where issues such as malnutrition, poverty, and the rising costs of animal proteins are already pressing concerns [4].

Insects represent one of the most diverse groups in the animal kingdom, with an estimated 5.5 million species in the class Insecta, of which only about one million have been identified [5]. Historically, entomophagy—the practice of consuming insects—has been a common dietary component in many cultures, particularly in regions of Asia, Africa, and Latin America [6,7]. Globally, over 1900 edible insect species have been identified, with Mexico, China, Thailand, and India among the countries with the highest diversity of edible insects [8]. In Mexico alone, more than 100 species of edible insects have been consumed since pre-Hispanic times, including representatives from orders such as Coleoptera (31%), Lepidoptera (18%), Hymenoptera (14%), Orthoptera (13%), and Hemiptera (10%) [9,10].

Interest in insect consumption has been driven by their high nutritional value, environmental benefits, and potential to support the socio-economic development of communities [8]. In recent years, insect farming has been proposed as a solution to global protein shortages, both for human consumption and as feed for livestock [11]. This practice is increasingly recognized for its role in enhancing food security and sustainability (FSS) by providing an alternative protein source with a lower environmental impact than traditional livestock production [12]. The FAO highlights that edible insects can play a crucial role in addressing nutritional deficiencies, particularly in vulnerable populations, thereby strengthening food security [13]. Furthermore, edible insects align with the four pillars of food security—availability, access, utilization, and stability—by offering an accessible source of nutrition [14]. This makes them a viable option for meeting dietary needs while promoting equitable FSS [15].

Beyond their global significance, edible insects in Mexico serve a dual role as both cultural heritage and an emerging sector within the agri-food industry. Oaxaca in particular is renowned for its deep-rooted entomophagy traditions, featuring species such as grasshoppers (*Sphenarium purpurascens*, locally known as “chapulines”), leafcutter ants (*Atta mexicana,* referred to as “chicatanas”), and red agave worms (*Comadia redtenbacheri* Hamm. syn. *Hypopta agavis,* commonly called “gusanitos rojos de maguey”). These insects are not only integral to local traditions but are also gaining commercial traction in both domestic and international markets. However, industry growth is hindered by challenges such as the need for stringent biosafety protocols, regulatory frameworks, and standardized production practices to align with global food safety standards.

Research on the nutritional composition of edible insect-based products has highlighted significant variability, influenced by factors such as species, developmental stage, habitat, diet, and processing techniques [16]. For instance, adult house crickets (*Acheta domesticus*) have been shown to contain high levels of calcium, potassium, sodium, and magnesium in products from regions like the Czech Republic, Austria, and France. Yellow mealworm larvae (*Tenebrio molitor*) are distinguished by their high phosphorus content, while buffalo worm larvae (*Alphitobius diaperinus*) have been reported to exhibit lower levels of calcium, sodium, and magnesium, particularly in chocolate-based products from Austria. Similarly, domesticated silkworm pupae (*Bombyx mori*) in soy sauce-based products from South Korea have been reported to contain the lowest potassium and phosphorus levels. These findings underscore the importance of understanding the interplay between biological and environmental factors in determining the nutritional profiles of edible insects [16].

Despite their nutritional advantages, the study highlighted toxicological concerns. Products derived from *T. molitor* for animal feed contained the highest levels of aluminum, arsenic, cadmium, and lead, while *A. diaperinus* used in protein powders showed elevated nickel levels. These findings emphasize the importance of understanding how factors such as developmental stage, diet, habitat, and processing methods affect the nutritional and safety profiles of edible insects.

Edible insects remain highly nutritious, providing proteins, healthy fats (mono- and polyunsaturated fatty acids), and essential minerals such as iron, zinc, magnesium, and selenium [17,18,19,20]. Their versatility makes them an excellent option for both human diets and livestock feed. For humans, edible insects provide a sustainable and nutrient-rich alternative to traditional protein sources, addressing the needs of regions with limited access to animal proteins while meeting the growing demand for eco-friendly and resource-efficient food solutions. Additionally, their low environmental footprint and ability to transform organic waste into high-value products further reinforce their role as a cornerstone of FSS [21].

This study aims to fill knowledge gaps by examining global research trends on edible insects from 2009 to 2023, with a particular focus on biosafety, while also exploring the commercialization of grasshoppers in Oaxaca, Mexico. Using bibliometric tools, the research identifies influential authors and institutions, emerging topics, and the conceptual structure of the field, providing insights into its intellectual framework [22,23].

Furthermore, this study explores the challenges and opportunities for integrating edible insects into sustainable food systems in Mexico. By focusing on regulatory frameworks, production practices, and cultural acceptance, it seeks to promote FSS with the incorporation of edible insects into human diets, contributing to the development of effective strategies for their adoption at both local and global levels.

To provide a comprehensive analysis, this study is structured as follows: Section 2 details the methodology, including the bibliometric approach and the exploration of edible insect commercialization in Oaxaca, Mexico. Section 3 presents the results and discussion, highlighting global research trends, key contributors, and the challenges and opportunities in the edible insect industry. Section 4 concludes the study by summarizing key findings, addressing limitations, and proposing future research directions.

## 2. Materials and Methods

This study primarily employed a quantitative methodology, focusing on the analysis of bibliometric data [24] extracted from scientific publications on edible insects. Quantitative indicators, such as publication trends, citation metrics, and keyword co-occurrences, were analyzed using specialized bibliometric tools to identify influential authors, institutions, and emerging research themes.

### 2.1. Bibliometric Analysis

This bibliometric analysis was conducted using the Scopus database, a widely recognized platform known for its extensive coverage of multidisciplinary scientific publications. Data were processed and visualized using VOSviewer 1.6.20 [25] and Bibliometrix 4.1.2 [26], tools that enabled the visualization of interconnections among authors, keywords, and the most prolific research-producing countries. The keyword co-occurrence analysis was refined by eliminating low-relevance and infrequent keywords, setting a threshold of five occurrences. This approach enabled a deeper understanding of global research dynamics and trends within the field of edible insect studies.

### 2.2. Search Criteria, Exclusion, and Validation

Advanced search terms combined with Boolean operators “OR” and “AND” were employed to refine the search query. Key phrases such as “edible insect”, “entomophagy”, “biosecurity”, and “food safety” were applied to retrieve relevant publications. The search period spanned 2009 to 2023, as the year 2024 did not yet have a sufficient number of citations for inclusion.

**Figure 1:**
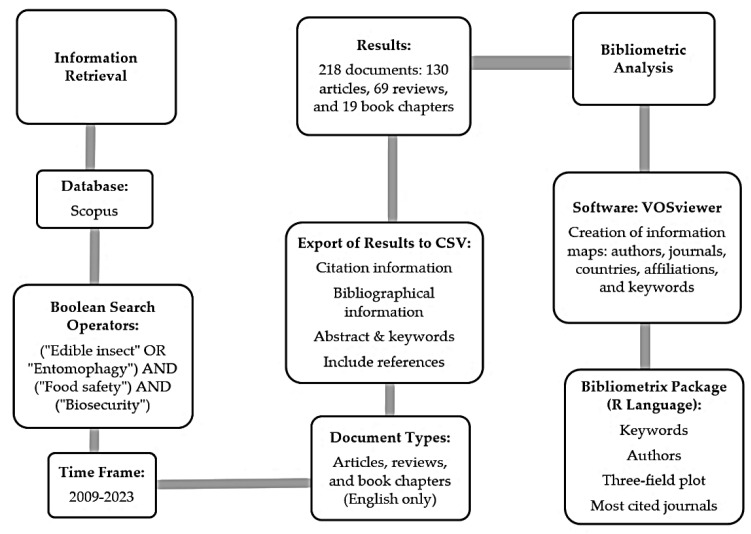
Flow diagram of the methodological process for bibliometric analysis.

To ensure the relevance and reliability of the collected literature, inclusion criteria were limited to scientific articles, reviews, and book chapters. Notes and complete books were excluded. To ensure consistency and comparability across the dataset, only documents published in English were included in the analysis. We manually eliminated duplicates to avoid repetition. This rigorous approach bolstered the validity of the bibliometric analysis (Figure 1).

In addition to the bibliometric analysis, the study explored the commercialization of edible insects in Oaxaca, Mexico, focusing on both virtual platforms and local markets. The investigation aimed to identify the types of insects utilized, the range of products offered, and their intended consumer groups. Data collection encompassed:

- Virtual platforms: Online stores specializing in edible insects for human and animal consumption were analyzed. Selection criteria included platform relevance, product availability, and consumer accessibility. Key data points collected comprised product type (whole insects, flours, snacks, or supplements), insect species used, pricing, and target consumers (human or animal feed).
- Local markets: Field visits were conducted in Oaxaca’s primary weekly and permanent markets to assess the availability, pricing, and diversity of edible insect-based products. Markets were selected based on their regional significance and volume of commercial activity. Observations focused on product presentation (raw, dried, seasoned, or processed), price variations across vendors, and consumer demand.

This mixed-methods approach offered a comprehensive perspective on the commercialization landscape, bridging global research trends with regional practices and consumer preferences. By integrating these elements, the study highlights the challenges and opportunities for the edible insect industry, particularly in the Mexican context.

## 3. Results and Discussion

### 3.1. Trends and Contributions in Global Edible Insect Research

**Figure 2:**
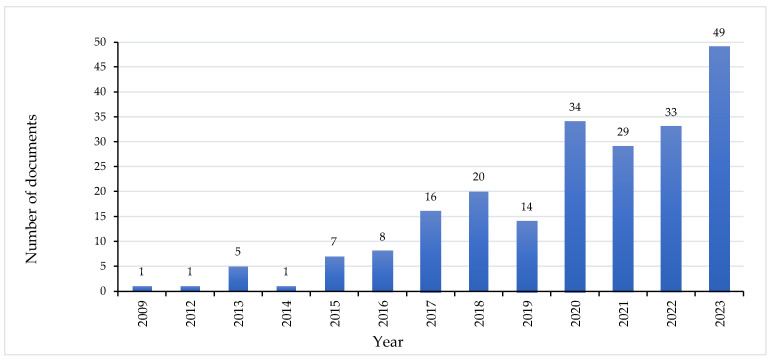
Global trends in edible insect research: Annual document publications (2009–2023).

The bibliometric analysis yielded a total of 218 documents, demonstrating a consistent increase in research output over the years (Figure 2). In 2023 alone, 49 documents were published, including 55.1% scientific articles, 34.7% review papers, and 10.2% book chapters. This steady growth reflects the expanding global interest in edible insect research and its potential contributions to FSS and biosecurity.

Countries leading in edible insect research, particularly with a focus on biosecurity, include Italy, Germany, the Netherlands, and Belgium. These nations are not only prominent in terms of research output—Italy with 42 documents, Germany (29), the Netherlands (25), and Belgium (21)—but also lead in citation impact, reflecting their contributions to advancing key themes such as nutritional value, food safety, and environmental impact. Citation metrics underscore Germany as the highest-ranking country with a score of 86.51, followed by the Netherlands (73.76), Brazil (52.66), and Belgium (48.09). These disparities underscore variations in investment in research and development, as well as the capacity to produce high-impact and widely referenced work (Table 1).

Developed countries exhibit greater expertise in adopting emerging technologies and focus their efforts on tackling critical challenges in edible insect research. This focus underscores their ability to advance the field through collaboration, innovation, and the effective application of knowledge. Additionally, the geographic distribution of research reveals a strong collaborative nature, further amplifying the impact of these nations on the global edible insect research landscape.

The first article, published in 2009 and titled “Edible insects: Traditional knowledge or western phobia?” [27], addressed the challenges posed by population growth and environmental degradation in providing sufficient animal protein. While many traditional cultures have long consumed insects as a protein source, Western societies remain resistant, despite their high meat consumption. The article proposed incorporating insects into diets as a means of reducing environmental footprints, with the key challenges identified as the development of sustainable production systems, fostering cultural change in Western societies, and ensuring environmental preservation.

In early 2023, the article “Insects as an alternative protein source for poultry nutrition: a review” [28] focused on the sustainability of insect-based diets for poultry. The study analyzed various species, including the black soldier fly (*Hermetia illucens*), housefly (*Musca domestica*), beetles (*Spodoptera littoralis*), *T. molitor*, *B. mori*, earthworms (*Eisenia fetida* and *Lumbricus terrestris*), crickets (*Acheta* sp.), and grasshoppers (Orthoptera: *Schistocerca* sp.), as alternative protein sources. This research signifies a transition from general discussions about the potential of edible insects for global human nutrition to more specialized and technical investigations. The progression of research in this field is further reflected in the contributions of key journals, affiliations, and authors, highlighting the growing global interest in edible insects (Table 2).

**Figure 3:**
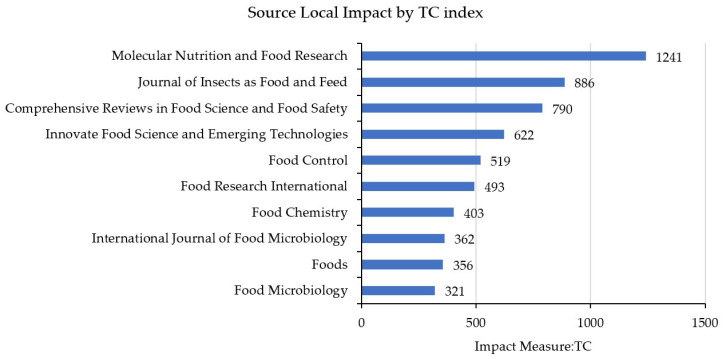
Most active journals in edible insect research based on citation count.

The journals with the highest visibility, links, and citations are: “Journal of Insects as Food and Feed” with 26 documents and 886 citations; followed by “Foods”, with 16 documents and 356 citations; “Food Research International” with 7 documents and 493 citations; and the “International Journal of Food Microbiology” with 6 documents and 362 citations. However, the journal with the highest overall impact (Figure 3) is “Molecular Nutrition and Food Research”, which has accumulated a total of 1241 citations, with its most significant article being “Nutritional composition and safety aspects of edible insects” [18].

The prominence of these specific journals reflects the increasing academic and industrial interest in edible insects, particularly in relation to their nutritional value and their potential to enhance global food security. The geographic distribution of research further emphasizes this trend, with leading countries in edible insect studies contributing significantly to the field’s advancement.

**Figure 4:**
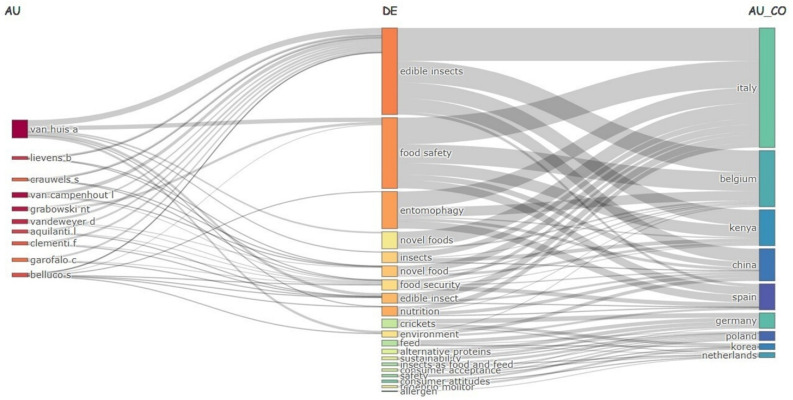
Three-field network mapping of authors, keywords, and countries in edible insect research.

The results (Figure 4) further illustrate this dynamic, showcasing the top 10 most influential authors, the 20 most frequently used keywords, and the 10 countries with which they collaborated. Notably, all authors were associated with the term “edible insects,” underscoring its central role as a unifying theme in this field and reflecting the collaborative and multidisciplinary nature of the research landscape.

The keyword analysis identified terms such as “food safety”, “entomophagy”, and “nutrition”, reflecting a strong interest within the scientific community in studying food safety and the nutritional value of insects. The research is notably concentrated in countries such as Italy, Belgium, Kenya, and China. This concentration may be explained by the significant interest in these countries regarding edible insects’ potential. This broader perspective is reflected in the analysis of keywords, which highlights the dominant themes, connections, and emerging trends in edible insect studies.

### 3.2. Keyword Analysis and Thematic Trends in Edible Insect Research

**Figure 5:**
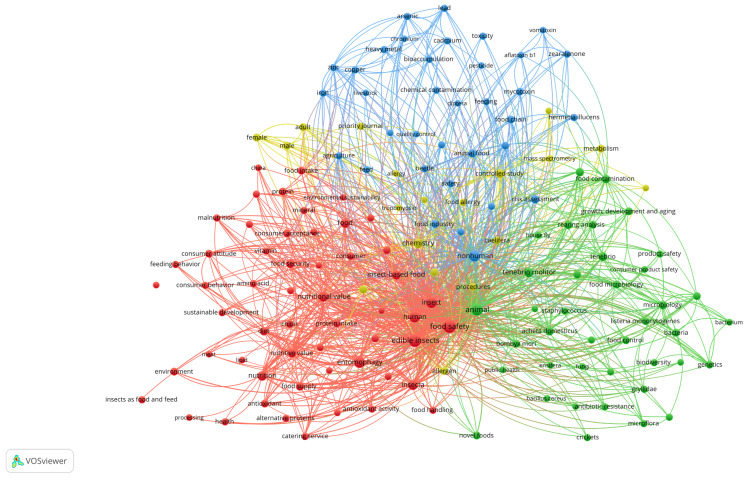
Keyword co-occurrence network: thematic clusters in edible insect research.

The analysis revealed that 166 keywords, from an initial set of 1941, were frequently used, appearing in at least five documents. These keywords were mapped to highlight the most commonly used terms in edible insect research. Using VOSviewer 1.6.20, the keywords were grouped into four distinct clusters (Figure 5), with each cluster representing a thematic axis. This grouping provided insights into the main research areas, connections, and emerging trends in the field. The keyword “food safety” emerged as the most prominent, appearing 142 times, underscoring its central role and extensive connections with other terms. It was followed by “edible insects” (112) and “entomophagy” (58), both demonstrating strong link strengths exceeding 300. These findings highlight the dominant themes and the interconnected nature of edible insect research.

The thematic clusters identified by the analysis provide further insights into the focus areas of edible insect research:

- Red cluster: This cluster addresses terms related to food safety, nutrition, and consumer acceptance of edible insects, incorporating references to regions like Africa and China. Prominent nodes include “food safety”, “edible insect”, “entomophagy”, “nutritional value”, “insect-based food”, “food security”, “protein”, “food processing”, “consumer acceptance”, “chitin”, “environmental impact”, “Africa”, “antioxidant activity”, “food insecurity”, “malnutrition”, “sustainability”, “sustainable development”, “alternative proteins”, “chemical hazards”, “insect farming”, “allergenicity”, “China”, and “trace element”.
- Green cluster: This cluster focuses on microbiology and food safety of edible insects, highlighting keywords such as “food microbiology”, “microflora”, and “bacterium”. These terms reflect the interest in microorganisms present in edible insects and their food safety risks. Additional terms such as “*Listeria monocytogenes”*, “*Staphylococcus”*, “*Salmonella*”, “*Bacillus cereus”*, “bacteria”, and “Enterobacteriaceae” underscore microbial hazards. The term “antibiotic resistance” suggests evaluations of antibiotic resistance in bacteria from insects. Additionally, species like “*Tenebrio molitor*”, “*Acheta domesticus*”, “*Bombyx mori*”, “grasshoppers”, and “*Musca domestica*” have also been studied, highlighting the biological diversity implications of edible insects, as indicated by the node “biodiversity”.
- Blue cluster: This cluster includes terms associated with biotechnology and processing, such as “quality control”, “food industry”, “chemical contamination”, and “bioaccumulation”. It also highlights safety concerns related to “pesticides”, “heavy metals”, and “contaminants”, emphasizing the importance of research in processing technologies, nutritional components, and food safety with regard to toxic substances and chemical residues.
- Yellow cluster: This cluster centers on food allergy and insect chemistry, featuring keywords like “adult”, “allergen”, “arginine kinase”, “Caelifera”, “chemistry”, “food allergy”, “hexapoda”, “mass spectrometry”, “metabolism”, “procedures”, and “tropomyosin”. These terms underscore the importance of identifying potential allergens in edible insects and developing precise methods for detection and analysis. The focus on “mass spectrometry” and “limit of detection” reflects an emphasis on advanced techniques for identifying and quantifying specific compounds in insects.

**Figure 6:**
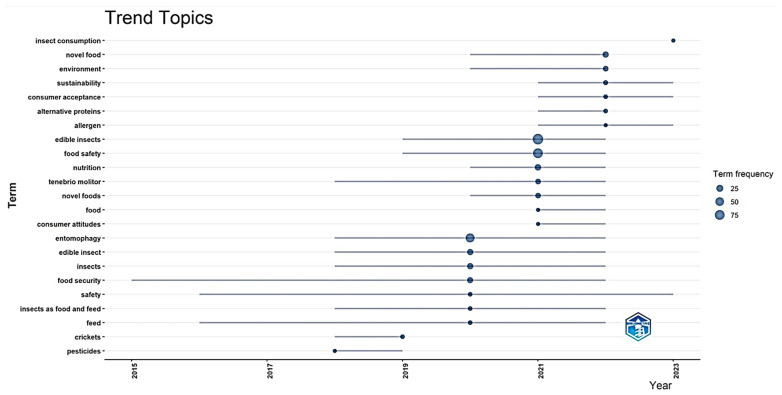
Temporal trends of key topics and keyword frequency in edible insect research.

The temporal distribution and frequency of keywords provide deeper insight into the evolution of edible insect research. Between 2015 and 2023 (Figure 6), foundational topics such as “food security” and “insect consumption” were more prominent in the early years, reflecting initial discussions on the role of edible insects in addressing global nutritional challenges. Over time, the research focus gradually shifted toward more specialized topics, including “*Tenebrio molitor*”, “nutrition”, and “food safety”, indicating a transition from broad conceptual discussions on entomophagy to more technical and applied research.

The keyword co-occurrence analysis (Figure 6) highlights dominant terms through larger circles, with “food safety”, “edible insects”, and “sustainability” emerging as recurring trends throughout the analyzed period. The horizontal lines represent the timeline in which these terms were predominantly used, illustrating the continuity and transformation of research priorities.

Between 2020 and 2023, emerging terms such as “*Tenebrio molitor”*, “alternative proteins”, and “biosafety” gained prominence, suggesting a research transition toward species-specific studies, nutritional profiling, and regulatory challenges. This shift underscores a growing emphasis on standardizing production methods, ensuring food safety compliance, and addressing consumer perception barriers. Furthermore, the increasing focus on biosafety and regulatory frameworks aligns with the expanding commercialization of edible insects and the need to establish rigorous food safety standards.

These temporal trends suggest a research trajectory that is evolving from general entomophagy discussions toward a more structured and regulation-driven approach. The progression of research topics reflects the need to harmonize scientific advancements with policy development to support the broader adoption of edible insects in global food systems.

### 3.3. Author Networks and Citation Impact in Edible Insect Studies

The results identify key authors who have significantly influenced the field of edible insect research, as identified through bibliographic coupling analysis. This analysis reveals established collaboration and citation patterns, as well as connections to emerging research areas, offering valuable insights into the intellectual structure of the field.

**Figure 7:**
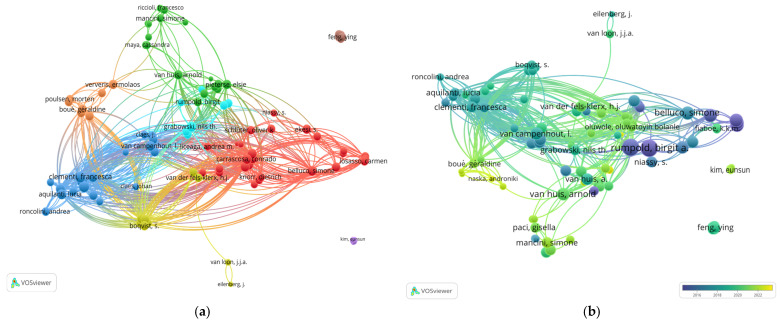
Collaboration and citation patterns in edible insect research: (**a**) Bibliographic coupling network of authors where colors represent distinct research clusters based on shared citations. Each color corresponds to a group of authors whose work is strongly linked through bibliographic coupling, indicating thematic research communities. (**b**) Temporal evolution of bibliographic coupling.

In terms of citation impact, larger nodes in the bibliographic coupling network represent authors with the highest number of citations. These documents are widely recognized and frequently referenced by multiple research groups, underscoring their importance in advancing knowledge on edible insects and food biosecurity (Figure 7). The color gradient of the nodes, ranging from dark blue for publications from 2016 to yellow for those from 2022, illustrates the evolution of collaborative networks over time and highlights the researchers who have been most active during specific periods.

**Figure 8:**
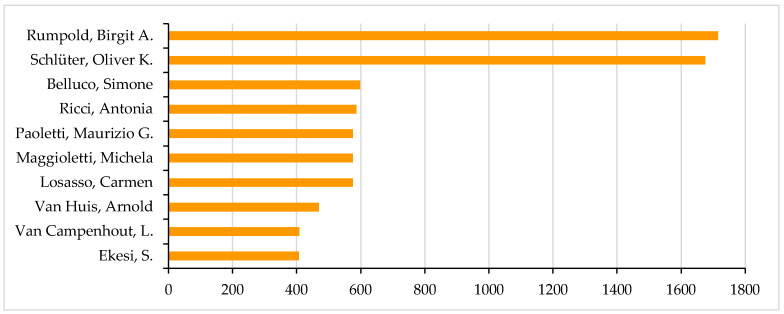
Authors with the Highest Citation Counts.

These relationships further highlight the influence of specific authors, with Rumpold, Birgit A. leading the citation count with 1715 citations, followed by Schlüter, Oliver K. with 1675 citations. Their contributions have significantly shaped the field, influencing current research directions and setting priorities for future investigations (Figure 8).

Moreover, highly cited articles offer valuable insights into both foundational and emerging research areas within the field. These publications address critical themes such as sustainability and biosecurity, serving not only as benchmarks for ongoing studies but also as guiding frameworks for future exploration. Their impact underscores the interconnected nature of key researchers and their pivotal role in advancing the field of edible insect research.

The importance of these contributions is further emphasized by the 10 most cited articles (Table 3), which provide a comprehensive overview of the potential of edible insects as an alternative source of food and feed. These studies address key aspects such as nutrition, food safety, production, and cultural acceptance, serving as foundational references that highlight both the benefits and challenges of integrating edible insects into global food systems. Together, they reflect the field’s evolution and its capacity to tackle pressing FSS issues.

The most cited article, *“*Nutritional composition and safety aspects of edible insects*”* [18], synthesized data from 236 studies on the nutritional composition of various edible insects. It examined essential components such as amino acids, fatty acids, minerals, and vitamins, highlighting both the benefits and risks of insect consumption. The study emphasized that edible insects, a traditional food source in many cultures, are highly nutritious, offering substantial protein content, healthy fatty acids, and micronutrients such as iron, magnesium, and zinc [18].

The second most cited article similarly recognized insects as a nutrient-rich alternative for both human and animal consumption. However, it placed significant emphasis on advancing rearing and processing technologies to ensure food safety and consumer acceptance on an industrial scale [19]. This underscores the growing recognition of edible insects as a solution to FSS challenges, while also acknowledging the technical and social barriers that must be addressed for broader adoption.

In general, the articles examined in the bibliometric analysis consider edible insects to be highly nutritious, providing elevated levels of proteins, healthy fats, essential vitamins, and minerals, making them an attractive option for both human diets and animal feed. They are particularly rich in amino acids and healthy fatty acids, such as monounsaturated and polyunsaturated fats, which form key elements of their nutritional profile [17,18,19]. Additionally, the analyzed literature highlights their significant amounts of minerals like iron, zinc, magnesium, and selenium, suggesting that insects could serve as an alternative to traditional animal protein sources [17,116].

However, while the nutritional potential of edible insects is well-documented [18,55], concerns remain regarding food safety, particularly concerning heavy metal contamination and biosafety standards, which must be managed effectively through methods such as thermal treatments, lactic fermentation, or drying processes. These challenges, as noted in the literature, necessitate effective management through processing techniques such as thermal treatments, lactic fermentation, or drying, which have been reported to enhance food safety while extending shelf life [18,55]. Additionally, improving processing methods is considered essential for mitigating microbiological risks and ensuring contaminant-free products, particularly in large-scale farming operations. The lack of clear legislation and regulatory frameworks in many countries is frequently cited as a significant barrier to the broader acceptance of edible insects in conventional markets [49,151].

From an environmental and economic perspective, the reviewed studies identify edible insects as a viable alternative to conventional livestock farming, emphasizing their minimal resource requirements and ability to convert organic waste into high-value food products. For instance, species such as *H. illucens* have demonstrated remarkable efficiency in bioconversion, contributing to organic waste management and feed production [49]. These qualities not only reduce environmental impact but also represent viable economic opportunities, particularly in regions facing acute food security challenges [15,39].

While edible insects hold great potential for food security and sustainability (FSS), their successful integration into mainstream markets requires addressing key challenges such as food safety, consumer acceptance, and regulatory frameworks. Scientific research plays a pivotal role in navigating these complexities, particularly in regions where entomophagy is deeply ingrained. Understanding regional research efforts can provide valuable insights into how scientific advancements align with local traditions and commercial opportunities.

### 3.4. Research Dynamics and Collaboration Networks in Edible Insect Studies in Mexico

Building on the global perspective, the Mexican context provides valuable insights into the increasing focus on edible insect research. Although relatively recent, research activity in this field in Mexico has shown notable growth since its inception in 2020. By 2023, six documents had been published, with 66.7% being review papers and 33.3% scientific articles. This trend underscores the country’s growing interest in the potential of edible insects, both from a scientific and practical perspective.

Among the key contributions to this field, the journal “Frontiers in Nutrition” stands out with 54 citations, followed by “Food Research International” and “Insects”, each with 40 citations. Additionally, the journal “Foods” published the most recent article in 2023, emphasizing its relevance in current research and its role in advancing the understanding of edible insects in the Mexican context. Together, these publications reflect Mexico’s increasing engagement with edible insect research, aligning with global trends while offering a regional perspective on the challenges and opportunities in this emerging field (Table 4).

**Figure 9:**
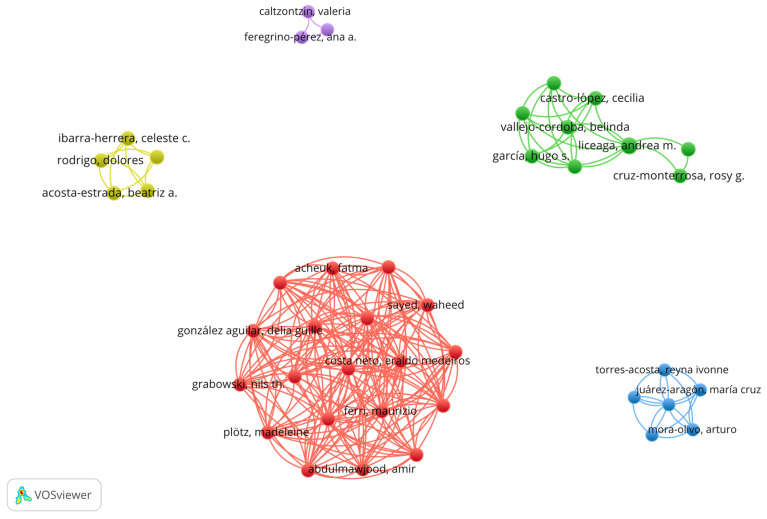
Collaboration network among Mexican authors in edible insect research.

This growing interest aligns with global trends, highlighting the expanding scope and relevance of edible insect studies in Mexico. Beyond journal citations, collaboration networks among Mexican authors reveal distinct patterns of co-authorship (Figure 9).

In these networks, different groups of authors are connected, representing collaborative relationships. When focusing on the number of documents produced, the green cluster stands out, with Liceaga, A. leading with two published documents. In contrast, the other clusters—red, blue, yellow, and purple—comprise authors with a single publication each. This suggests that the green cluster demonstrates more intense research activity, followed by the other clusters in descending order of collaboration and academic output.

The key contributions of these collaborative efforts highlight the most cited publications on edible insects in Mexico. These works have played a pivotal role in advancing the understanding of edible insects as a food source and their relevance to FSS. The listed studies (Table A1) reflect the growing attention toward harnessing insects with an emphasis on their nutritional value, technological applications, and food safety aspects. One innovative aspect explored in these studies is insect fermentation, a technique that has the potential to enhance their nutritional properties and create novel food products. This method is seen as a promising tool for the future development of the edible insect industry [94].

However, discussions about the safety of insects as a food source remain complex. Key determinants of their viability as a safe and environmentally responsible food include species life cycles, processing methods, and consumer acceptance [21]. Field studies have also highlighted challenges associated with the collection and processing of insects, such as grasshoppers, where the presence of accompanying arthropods and plant debris could compromise product safety. These findings underscore the need to optimize handling practices [152]. Conversely, aquatic insect eggs, such as those from the genus Corixidae, have been shown to be a rich source of phenolic compounds, antioxidants, and fatty acids. This reinforces the potential of insects as nutrient-dense foods with health benefits [9].

While research on edible insects in Mexico continues to expand, its practical impact depends on how these findings translate into real-world applications. Beyond scientific advancements, the successful commercialization of edible insects hinges on consumer attitudes and market dynamics, requiring a deeper exploration of the sociocultural and economic factors that shape their adoption.

### 3.5. Consumer Acceptance, Sustainability, and International Regulations

Despite the growing global interest in edible insects, several obstacles hinder their widespread adoption, including cultural biases, safety concerns, and complex regulatory landscapes [153]. While entomophagy is widely practiced and accepted in many regions of Africa [39], Western societies often view insect consumption with aversion [27], perceiving it as unappealing, primitive, or associated with poverty [10,15,17]. Psychological factors such as food neophobia and the ‘disgust factor’ contribute significantly to resistance, as insects are frequently linked to dirt, disease, and mortality [153]. However, research suggests that exposure, strategic branding, and product innovation can positively influence consumer perception [154,155].

Recent consumer studies highlight that familiarity with insect-based products plays a crucial role in acceptance. For instance, rebranding insects as “protein-rich superfoods” and integrating them into processed food formats (e.g., pasta, protein bars, and snacks) has significantly increased consumer willingness to try these products in European markets [156,157,158,159]. Furthermore, demographic factors play a role in consumer willingness to adopt edible insects. Research indicates that younger men, particularly those with lower meat consumption and higher environmental awareness, are more open to incorporating insects into their diets [16,160]. However, their acceptance remains contingent on presentation, product labeling, and clear information regarding nutritional benefits and sustainability claims [161]. These findings suggest that targeted educational campaigns and innovative product development are essential in overcoming psychological and cultural barriers to insect consumption.

From a sustainability perspective, edible insects offer promising solutions to address the growing demand for protein driven by population growth and the escalating costs of traditional animal-based products [2,162]. Unlike conventional livestock farming, which requires substantial land, feed, and water resources, insects exhibit high reproductive rates, minimal spatial requirements, and omnivorous diets. Additionally, their production generates a significantly smaller carbon footprint, with meat production alone accounting for over 7.1 billion tonnes of CO₂ equivalents annually—14.5% of all anthropogenic greenhouse gas emissions [163,164]. Insects’ ability to convert organic waste into high-value protein further positions them as a resource-efficient and environmentally friendly alternative to traditional protein sources [18]. In this context, it is also important to consider alternative protein sources such as bee farming. Despite being consumed in various tropical regions, further research into honey-producing bee species is required to better understand their functional properties and potential applications [11].

Regulatory frameworks play a critical role in determining the scalability and market viability of edible insects. However, global regulations remain fragmented, with significant differences between the European Union (EU), the United States (US), and Latin American countries, including Mexico.

The EU has established some of the most stringent standards, particularly regarding mycotoxin contamination. Fungal contaminants such as *Aspergillus*, *Penicillium*, and *Fusarium* are rigorously monitored, with maximum permissible levels for Aflatoxin M1 set between 0.025 and 0.05 μg/kg, and Ochratoxin A between 0.5 and 5 μg/kg. By comparison, countries like the United States and China allow higher thresholds, while Brazil lacks defined limits for certain mycotoxins, including Ochratoxin A and Deoxynivalenol [165].

A notable regulatory milestone was achieved in January 2023 when the EU approved the commercialization of defatted *A. domesticus* (house cricket) powder, produced by Cricket One Co. Ltd., located in Ho Chi Minh City, Vietnam. This approval, valid for five years, followed a rigorous evaluation by the European Food Safety Authority (EFSA), allowing its use in various food applications (e.g., bread, pasta, sauces, snacks, and meat substitutes). However, allergic risks have been noted, particularly for individuals allergic to crustaceans, mollusks, or dust mites. The EU also imposed strict mycotoxin limits, setting Aflatoxins (B1, B2, G1, G2) at ≤0.4 μg/kg, Deoxynivalenol at ≤200 μg/kg, and Ochratoxin A at ≤1.0 μg/kg [166].

Similarly, regulatory progress is extending to other insect species, such as *A. diaperinus*. Ynsect NL BV, based in Ermelo, The Netherlands, has applied for EU approval for its commercialization, proposing its use in frozen, dried, or powdered forms in various food applications, including dietary supplements. Like *A. domesticus*, strict mycotoxin limits have been set to ensure safety [166,167] (Table 5).

To unlock the full potential of Mexico’s edible insect industry, policymakers must establish biosafety standards, product certification, and regulatory frameworks that align with global food safety requirements while accommodating local market dynamics. Drawing from structured models like the EU’s regulatory approach [166,167], Mexico can develop policies that enable safe commercialization, boost consumer confidence, and enhance international market access [168].

Beyond scientific and regulatory considerations, the successful integration of edible insects into mainstream markets requires a comprehensive approach that addresses supply chain dynamics, consumer perception, and commercialization strategies.

### 3.6. Challenges and Opportunities in Insect Farming and Commercialization in Mexico

Mexico, known for its deep-rooted tradition of entomophagy [169], is experiencing substantial growth in the farming and commercialization of edible insects for both human and animal consumption [169]. This practice, particularly the consumption of grasshoppers (*S. purpurascens,* locally known as “chapulines”), leafcutter ants (*A. mexicana*, referred to as “chicatanas”), and red agave worms (*C. redtenbacheri*, commonly called “gusanitos rojos de maguey”), is deeply rooted in Oaxaca, Mexico’s, cultural heritage and forms an integral part of its intangible cultural patrimony [170]. The tradition of insect consumption is strongly tied to the transmission of ancestral knowledge from one generation to the next, emphasizing the nutritional value of insects, particularly their high protein content, as a dietary staple since pre-Hispanic times [171,172].

Despite this rich cultural heritage, modern dietary shifts in Mexico, including an increased preference for fast food, have led to a significant rise in health issues. Over 70% of individuals aged 20 and above are now reported to be overweight or obese [171]. In contrast, the consumption of insect-based foods has been gaining momentum in European countries due to cultural globalization [3,19]. This phenomenon is reshaping dietary habits and increasing awareness of insect-based foods as public health indicators. However, in urban areas of Mexico, sociocultural dynamics often lead minority groups to adopt the dietary habits of dominant urban cultures, resulting in a gradual decline in the consumption of traditional pre-Hispanic foods and contributing to a sense of cultural detachment or even shame associated with such practices [171].

Encouraging the consumption of edible insects is essential not only to preserve Mexico’s cultural heritage but also to address health and sustainability challenges. Promoting traditional foods like chapulines can improve public health outcomes, strengthen indigenous traditions, and foster cultural pride [170]. Gastronomic initiatives centered around chapulines further position them as a “superfood,” highlighting their potential as a sustainable protein source for future generations. Such initiatives emphasize the significant social and health benefits of revitalizing insect consumption while preserving indigenous traditions and promoting healthier eating habits [173,174,175,176].

In Oaxaca, chapulines (*S. purpurascens*), chicatanas (*A. mexicana*), and red agave worms (*C. redtenbacheri*) remain staples in local and weekly markets. The latter markets, commonly referred to as “día de plaza,” are traditional open-air marketplaces held on specific days of the week in various towns and villages. These vibrant markets feature a diverse array of stalls offering locally produced goods, such as fresh produce, handmade crafts, traditional foods, and regional specialties. Prices for chapulines range from $2.50 to $3.00 USD per 120 g, depending on the product presentation and point of sale. Chicatanas, on the other hand, are available in various forms, with prices ranging from $6.00 USD for 45 g of salted ants to $20.00 USD for 150 g (Table A2). Additionally, red agave worms are sold raw or alive at $12.50 USD for 5 g. This accessibility not only reflects the region’s enduring culinary traditions but also aligns with the global trend toward sustainable protein sources [170,171,172,177].

The expansion of the edible insect industry in Mexico represents a fusion of cultural heritage and global demand. The industry has diversified its offerings to include species such as *A. domesticus*, *S. purpurascens*, *T. molitor*, *Zophobas morio*, and *H. illucens*, among others. These insects are processed into a wide array of products, including live insects, flours, snacks, and dietary supplements, meeting the demands of both human and animal nutrition. This diversity underscores the industry’s ability to meet varied market demands while preserving Mexico’s cultural identity and culinary traditions (Table 6).

These efforts should be reinforced by initiatives that strengthen local production chains, improve quality control, and integrate traditional knowledge into modern commercialization strategies [120,185,186]. Successfully addressing these barriers will enable Mexico to merge its entomophagy heritage with global trends, securing its position in the sustainable protein market while ensuring economic and cultural resilience [187].

At the same time, the expansion of the edible insect industry underscores the need for rigorous biosafety measures to ensure product safety for both human and animal consumption. Implementing such measures is essential to mitigating contamination risks and meeting the increasing global demand for insect-based products, which is expected to reach USD 8 billion by 2030. North America and Europe anticipate over 43% market growth by 2024, while the Asia–Pacific market is forecasted to exceed USD 270 million within the same period [188]. In Mexico, edible insect sales reached USD 50 million in 2022, with an estimated annual growth rate of 10%, reflecting rising demand both locally and internationally [168]. This trend highlights the opportunity for Mexico to strengthen its role in the global edible insect market while preserving its cultural and gastronomic heritage.

### 3.7. Balancing Innovation and Tradition in Industry Growth

**Figure 10:**
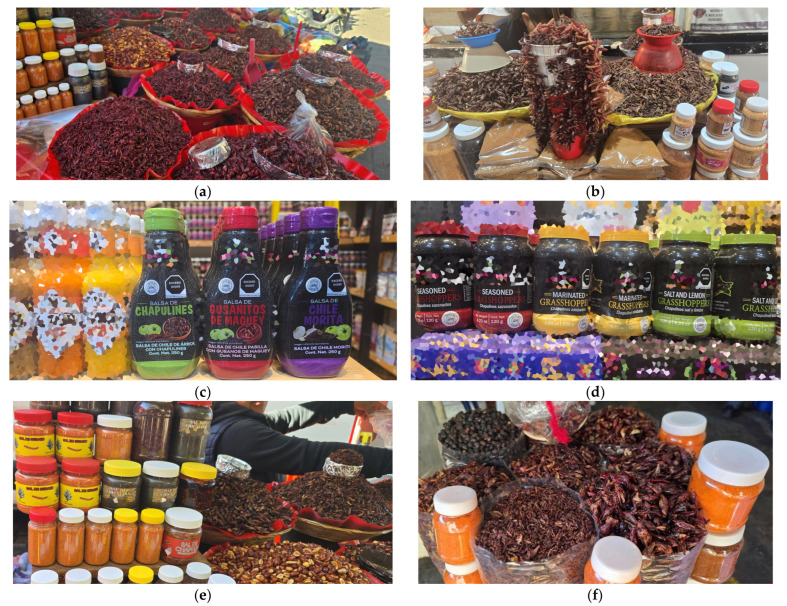
Edible insects in Oaxaca’s local markets: (**a**) Various chapulín presentations for sale. (**b**) Raw red agave worms, chapulines, and worm salt. (**c**) Spicy sauces with chapulines. (**d**) Bottled chapulines in different preparations. (**e**) Red agave worm salt, bottled ground chapulines, and bottled chicatanas. (**f**) Chicatanas, chapulines, and live or dried red agave worms.

As Mexico’s edible insect industry evolves, diversification plays a crucial role in catering to different consumer preferences while maintaining cultural authenticity. Globalization and increasing interest in Oaxaca’s rich culinary traditions are key drivers of this expansion. Tourists, captivated by the distinct flavors and cultural significance of edible insects such as chapulines (grasshoppers), frequently seek these products beyond their initial experience, contributing to their rising popularity in international markets (Figure 10).

However, achieving broader acceptance and ensuring safety necessitates continued advancements in research and regulation [168,169]. The identification and quantification of specific compounds in edible insects—such as nutrients, allergens, and contaminants—are crucial areas of focus (Table 7).

Ensuring food safety and regulatory compliance remains a pivotal challenge for the edible insect industry in Mexico [152,172]. As commercialization expands, establishing rigorous biosafety standards and improving research on contaminants and nutritional profiles will be essential to maintaining consumer confidence and meeting international market requirements.

### 3.8. Biosafety Challenges and the Global Perspective

Biosafety plays a crucial role in determining the scalability and market acceptance of edible insects. These organisms can be consumed at various life stages—egg, larva, pupa, and adult—with larvae and pupae being the most commonly consumed forms. Indirect consumption also occurs through products derived from or excreted by insects, often mixed with other ingredients [10].

Globally, leading companies such as Protifarm (Netherlands), AgriProtein (South Africa), Ynsect (France), and Flying Spark (Israel) are advancing insect farming. These enterprises focus on species like *H. illucens*, *T. molitor*, *A. domesticus*, and locusts for both human and animal use [2]. In Europe, insect-derived products for animal feed primarily utilize black soldier flies, yellow mealworms, and buffalo worms (*A. diaperinus*), while species like *G. mellonella*, *Bombix mori* (silkworm), grasshoppers, and crickets are widely employed for pet and zoo animal feed [4].

Despite these advancements, disease propagation in insect farming presents significant challenges. Insects like *A. domesticus* and *Gryllus assimilis* are commonly farmed for human and animal consumption, yet their microbiological composition varies based on feed, production conditions, and batches [193,194]. Pathogens such as *Salmonella*, *Listeria*, and *Klebsiella* spp. have been identified in farmed crickets, posing food safety concerns. While processing methods such as boiling and drying can inactivate bacterial pathogens, spore-forming microorganisms like *Clostridium* and *Bacillus* often resist these treatments [29,194].

Viruses further complicate the farming process. For instance, *A. domesticus* is susceptible to viral infections from families such as *Dicistroviridae*, *Iflaviridae*, *Iridoviridae*, *Parvoviridae*, and *Baculoviridae*, causing significant mortality. Similarly, the African migratory locust (*Locusta migratoria*), while valued as a food source, has been linked to agricultural damage and pathogens such as *Poxviridae* and *Reoviridae* [138,142].

While biosafety challenges remain a critical concern in insect farming, addressing these risks through improved safety protocols and disease monitoring is essential for industry expansion. As global markets increasingly integrate edible insects into food and feed supply chains, Mexico faces both opportunities and challenges in aligning its traditional practices with modern commercialization efforts.

### 3.9. Mexico’s Position in the Edible Insect Industry

Mexico’s edible insect sector reflects global trends, blending ancestral practices with contemporary market demands [169]. This fusion of cultural heritage and scientific innovation positions the country to contribute significantly to both local and international food security efforts [170]. However, to fully realize this potential, it is essential to address biosafety concerns, optimize farming practices, and establish rigorous safety standards that align with international regulations.

Despite growing domestic and international interest, the lack of formalized policies and standardized farming protocols remains a major barrier to industry expansion [168]. The absence of clear regulatory frameworks limits large-scale commercialization and restricts access to global markets. Strengthening traceability systems, certification programs, and food safety regulations would allow Mexico to develop a more structured and competitive edible insect sector while ensuring product quality and consumer trust [169].

Additionally, Mexico’s rich biodiversity and extensive entomophagy traditions provide a unique opportunity to diversify insect-based products beyond conventional species used in the global market [152,170,172]. As the sector evolves, continued investments in research, enhanced regulatory frameworks, and consumer education will be crucial for bridging the gap between tradition and commercialization.

## 4. Conclusions

The findings of this study reinforce the growing global recognition of edible insects as a sustainable and viable protein source, while highlighting Mexico—particularly Oaxaca—as a key region where entomophagy remains deeply embedded in cultural traditions. The bibliometric analysis confirms steady research growth worldwide, yet Mexico’s contributions remain limited, underscoring the need for greater research investment and stronger integration into the global scientific landscape. Despite its long-standing traditions, the commercialization of edible insects in Mexico is largely informal, hindered by the absence of standardized safety protocols and regulatory frameworks. These limitations pose significant challenges for expanding production and accessing international markets.

Overcoming these barriers requires a comprehensive approach that integrates scientific innovation with traditional practices. Establishing clear biosafety regulations, traceability systems, and quality standards is essential to ensure consumer safety and confidence. The adoption of advanced analytical techniques, such as LC-MS/MS and ICP-MS, can help detect contaminants and allergens, thereby facilitating compliance with international food safety requirements. Additionally, incentive programs and certification schemes could encourage producers to implement best practices, enhancing both domestic and global competitiveness.

This study has certain methodological limitations. The bibliometric analysis relied solely on the Scopus database, which, while extensive, does not cover all scientific publications, particularly those in non-English languages. As a result, valuable regional insights, especially from Latin America and Asia, may have been underrepresented. Similarly, the market analysis focused exclusively on Oaxaca, providing a localized perspective on commercialization trends that may not fully reflect the broader dynamics of Mexico’s edible insect industry. Future research should incorporate additional databases, multilingual sources, and expanded geographic coverage to achieve a more comprehensive understanding of the field.

From a managerial perspective, key stakeholders—including farmers, processors, and distributors—must standardize quality control measures, optimize supply chain logistics, and utilize digital platforms to increase market accessibility. Strengthening collaborations between research institutions, industry players, and policymakers will be critical in developing a sustainable and scalable edible insect industry that aligns with global food security strategies. Mexico has the potential to position itself as a leader in sustainable entomophagy, but achieving this requires targeted efforts to integrate cultural heritage with modern commercialization strategies while ensuring economic and environmental sustainability.

## Figures and Tables

**Table 1:** Leading countries in edible insect research: Number of publications, citations, and impact scores *.

Country	Documents	Citations	Score
Germany	29	32.2027	86.5172
Netherlands	25	38.7392	73.76
Brazil	6	9.839	52.6667
Belgium	21	22.9612	48.0952
Kenya	16	16.3938	46.4375
Italy	42	40.381	45.4048
Thailand	6	3.9526	41
China	10	12.5229	36.8
Poland	9	12.4427	30.3333
Uganda	5	2.5782	26.8
United States	16	14.3734	26.3125
Denmark	9	4.8112	22.6667
Spain	11	12.5223	22.5455
South Africa	9	6.9103	22.1111
Mexico	6	4.9133	21.6667
Portugal	10	6.4375	20
Nigeria	6	2.2648	19.5
Republic of Korea	6	4.2209	19.3333
France	8	5.7853	14.75
India	6	9.4452	11.5
Greece	5	2.6368	9.4

* Thresholds used in VOSviewer: minimum number of documents of a country = 5, total number of countries = 61.

**Table 2:** Top 10 journals, affiliations, and authors in edible insect research.

Id.	Journals (n = 82)		Affiliations (n = 91)		Authors (n = 65)	
1	Journal of Insects as Food and Feed (26)	[29,30,31,32,33,34,35,36,37,38,39,40,41,42,43,44,45,46,47,48,49,50,51,52,53,54]	Wageningen University & Research (19)	[15,33,40,49,50,55,56,57,58,59,60,61,62,63,64,65,66,67,68]	Van huis, Arnold. (11)	[15,49,50,61,62,63,64,65,66,67,68]
2	Foods (16)	[9,69,70,71,72,73,74,75,76,77,78,79,80,81,82,83]	KU Leuven (13)	[44,46,51,84,85,86,87,88,89,90,91,92,93]	Van Campenhout, L. (9)	[51,85,87,88,89,90,91,92,93]
3	Food Research International (7)	[58,85,94,95,96,97,98]	International Centre of Insect Physiology and Ecology Nairobi (11)	[30,39,41,45,99,100,101,102,103,104,105]	Grabowski, Nils Th. (7)	[21,106,107,108,109,110,111]
4	International Journal of Food Microbiology (6)	[89,90,92,109,112,113]	Tierärztliche Hochschule Hannover (9)	[21,106,107,108,109,110,111,114,115]	Vandeweyer, D. (7)	[46,51,85,89,90,91,92]
5	Comprehensive Reviews in Food Science and Food Safety (5)	[60,116,117,118,119]	Københavns Universitet (7)	[33,37,38,56,68,99,120]	Lievens, B. (6)	[87,88,89,90,91,93]
6	Critical Reviews in Food Science and Nutrition (5)	[103,105,121,122,123]	Leuvens Centrum voor Levensmiddelen en Voedingswetenschappen (7)	[51,85,87,88,89,90,93]	Aquilanti, L. (5)	[92,95,112,113,124]
7	Nutrients (5)	[125,126,127,128,129]	Faculteit Bio-igenieurswetenschappen (7)	[46,51,85,87,89,90,93]	Belluco, Simone. (5)	[60,97,116,130,131]
8	Animals (4)	[132,133,134,135]	Università Politecnica delle Marche (6)	[92,95,112,113,124,136]	Clementi, Francesca. (5)	[92,95,112,113,124]
9	Efsa Journal (5)	[137,138,139,140,141]	Istituto Zooprofilattico Sperimentale delle Venezie (6)	[60,97,116,130,131,142]	Crauwels, S. (5)	[87,88,89,90,93]
10	Food Control (4)	[55,143,144,145]	Autorità Europea per la Sicurezza Alimentare (6)	[34,146,147,148,149,150]	Garofalo, Cristiana. (5)	[92,95,112,113,124]

**Table 3:** Key publications in edible insect research ranked by citation count.

No	Title	Journal	TC	TCy
1	Nutritional composition and safety aspects of edible insects [18].	Molecular Nutrition and Food Research	1106	92.17
2	Potential and challenges of insects as an innovative source for food and feed production [19].	Innovative Food Science and Emerging Technologies	548	45.67
3	Edible insects in a food safety and nutritional perspective: A critical review [116].	Comprehensive Reviews in Food Safety	519	43.25
4	Microbiological aspects of processing and storage of edible insects [55].	Food Control	338	26.00
5	Insects as food and feed, a new emerging agricultural sector: A review [49].	Journal of Insects as Food and Feed	258	51.60
6	African edible insects for food and feed: Inventory, diversity, commonalities and contribution to food security [39].	Journal of Insects as Food and Feed	252	25.20
7	Edible insects: An alternative of nutritional, functional and bioactive compounds [17].	Food Chemistry	227	45.40
8	Edible insects: Traditional knowledge or western phobia? [27].	Entomological Research	219	13.69
9	Benefits and food safety concerns associated with consumption of edible insects [151].	NFS Journal	214	42.80
10	Edible insects contributing to food security? [15].	Agriculture and Food Security	184	18.40

TC = Total Citations; TCy = Total Citations per year.

**Table 4:** Edible insect research information in Mexico (2020–2023).

Journal	Year	Document	Citations
Frontiers in Nutrition	2021	1	54
Insects	2022	1	40
Food Research International	2020	1	40
Frontiers in Sustainable Food Systems	2022	1	11
Southwestern Entomologist	2020	1	1
Foods	2023	1	0

**Table 5:** Comparative Table: Edible Insect Regulations in Key Regions.

Regulation Aspect	European Union	United States	Mexico
Mycotoxin Limits	Strict (Aflatoxin ≤ 0.4 μg/kg, DON ≤ 200 μg/kg)	Higher thresholds	Undefined limits
Product Approval	*A. domesticus* & *A. diaperinus* approved for various food applications	No formal approval process for edible insects	No official approvals, mainly informal markets
Biosafety & Contaminants	Mandatory EFSA evaluation	Case-by-case approval	Lack of defined safety assessments
Labeling & Consumer Protection	Strict allergen labeling required	No specific regulations for insects	No official requirements for edible insect products
Market Integration	Structured regulatory pathways	Limited but expanding interest	Predominantly informal trade

**Table 6:** Edible insect-based food and feed products by Mexican companies.

Company	Insect Used	Product Sales	Target Species
Zofo [178], Quintana Roo.	*Z. morio* *S. purpurascens*	Worm Salt (dried chili, habanero lime, mango with chili). Baked Snack (fine herbs, habanero, sea salt, and lime).	Humans
Griyum [176], México.	*S. purpurascens*	Bulk Dehydrated Crickets	Humans
Tenebrios.com [179], Ciudad de México.	*T. molitor* *Z. morio* *Blaptica dubia* *Nauphoeta cinerea* *A. domesticus* *Blaberus giganteus*	Live	Reptiles, amphibians, fish, arthropods, birds, and small mammals
Insect nutrition [180], Guanajuato.	*A. domesticus* *H. illucens* *T. molitor*	Dehydrated for amphibians, fish, reptiles, birds, mammals Cricket Humus Cricket and Mealworm Flour	Reptiles, fish, birds, rodents, and hedgehogs
ReptileInk [181], Nuevo León.	*A. domesticus* *T. molitor* *Z. morio* *N. cinerea* *B. dubia* *Galleria mellonella*	Live	Animals
Illucens México [182], Mérida.	*H. illucens*	Biostimulant for crops and soils from Black Soldier Fly larval exuviae Black Soldier Fly Larvae Flour	Animals and plants
Grichosmx [175], México.	*A. domesticus* *T. molitor* *H. illucens*	Live	Reptiles, birds, and fish
Delinsect [173], Estado de México.	*Liometopum apicolatum* *S. purpurascens* *Aegiale hesperiasis* *C. redtenbacheri* *A. mexicana* *Thasus gigas*	Chicatana Macha Sauce Chapulín Macha Sauce Red Worm Salt Chapulín Salt Chapulín Flour	Humans
OptiProt [183], Morelos.	*T. molitor*	Flour Dehydrated	Humans
Gricha [174], Jalisco.	*S. purpurascens*	Snack (churros) Dehydrated and Flavored Whole Grain Cookies Proteins (chocolate, vanilla, coffee, and matcha) Flour	Humans
Erizos [184], Morelos.	*Z. morio* *A. domesticus* *T. molitor* *B. dubia* *N. cinerea* *Gromphadorhina portentosa*	Dehydrated Live	Arachnids, amphibians, rodents, birds, hedgehogs, fish, reptiles, and monkeys

**Table 7:** Techniques used for analyzing nutritional and contaminant profiles in edible insects *.

Title	Technique	Description	Findings
Evaluation of Hazardous Chemicals in Edible Insects and Insect-Based Foods for Human Consumption [189].	Organic chemical mass fraction analysis and non-targeted detection analysis	Investigation of flame retardants, PCB, DDT, dioxin compounds, pesticides, and metals (As, Cd, Co, Cr, Cu, Ni, Pb, Sn, Zn) in edible insects including *G. mellonella*, *L. migratoria*, *T. molitor*, *A. diaperinus*, and insect-based products in Belgium.	Organic chemical mass fractions and metal levels were relatively low, similar to those found in common animal products.
Identification of a Novel Allergen in Edible Insect *Gryllus bimaculatus* and Its Cross-Reactivity with *Macrobrachium* spp. Allergens [190].	SDS-PAGE (denaturing polyacrylamide gel electrophoresis) and IgE Immunoblotting with LC-MS/MS	Protein separation to identify allergens in *Macrobrachium* spp. and *G. bimaculatus* for food safety control and allergy diagnostics.	Arginine kinase (AK), glyceraldehyde 3-phosphate dehydrogenase (GAPDH), and hemocyanin (HC) were identified as allergens in *Macrobrachium* spp., while hexamerin1B (HEX1B) was identified as a novel allergen in *G. bimaculatus*. AK showed cross-reactivity between the two species.
Identification and Quantitative Analysis of β-Sitosterol Oxides in Oil from Two Varieties of Macrotermitinae in the Congo [191].	Gas chromatography (GC) and mass spectrometry (MS)	Analysis of larvae exposed to aflatoxin B1, deoxynivalenol, and zearalenone to determine mycotoxin concentration and accumulation in larval bodies.	No mycotoxin accumulation was detected in larval bodies, and exposure did not affect larval mortality or biomass.
Aflatoxin contamination detected in nutrient and anti-oxidant rich edible stink bug stored in recycled grain containers [102].	LC-Qtof-MS (liquid chromatography coupled with quadrupole time-of-flight mass spectrometry) and GC-MS (gas chromatography coupled with mass spectrometry)	Evaluation of mycotoxins, antioxidants, amino acids, and essential fatty acids in *Encosternum delegorguei* consumed in southern Africa.	Low levels of aflatoxin B1 were detected in insects stored under traditional conditions. Additionally, 10 fatty acids (7 essential), 4 flavonoids, and 12 amino acids (2 limiting in cereal-based diets) were identified. The stink bug is rich in proteins and fats but low in minerals, except for phosphorus.
Degradation and Excretion of the Fusarium Toxin Deoxynivalenol by Edible Yellow Mealworm (*T. molitor* L.) [59].	LC-MS/MS (liquid chromatography coupled with mass spectrometry)	Analysis of deoxynivalenol (DON) and its derivatives in *T. molitor* larvae and their feces to assess retention, sequestration, excretion, or detoxification of mycotoxins.	No DON or its derivatives were detected in harvested larvae, indicating potential degradation by the larvae. Approximately 14% of ingested DON in naturally contaminated diets and 41% in DON-enriched diets were excreted.
Effects of Mycotoxins Aflatoxin B1, Deoxynivalenol, and Zearalenone on Survival, Biomass, and Toxin Accumulation in *M. domestica* Larvae [57].	LC-MS/MS	Quantification of β-sitosterol oxides in sun-dried and smoked termites to evaluate compound concentrations in animal oils.	Termite oil contained higher concentrations of β-sitosterol oxides (29.1 μg.g-1 in sun-dried termites and 39.1 μg.g-1 in smoked termites) compared to edible vegetable oils.
Determination of Selected Elements in Two Commercially Available Edible Aquatic Insects (Coleoptera) and Their Updated Global Listing [192].	ICP-MS (inductively coupled plasma mass spectrometry)	Evaluation of mineral contents (Ca, Fe, Cu, Zn, Se, Co, Cr, As, and Pb) in two species of edible aquatic beetles (*Dytiscus marginalis* and *Cybister tripunctatus*).	Antinutritional elements (Pb and As) were detected in aquatic insects, but their levels were below toxic thresholds for humans.

* Advanced analytical techniques, including liquid chromatography-tandem mass spectrometry (LC-MS/MS) and inductively coupled plasma mass spectrometry (ICP-MS), facilitate the detection of mycotoxins, minerals, and other relevant compounds.

## Data Availability

The original contributions presented in this study are included in the article. Further inquiries can be directed to the corresponding authors.

## Abbreviations

The following abbreviations are used in this manuscript:

AK	Arginine kinase
DDT	Dichloro-diphenyl-trichloroethane
DON	Deoxynivalenol
EU	European Union
EFSA	European Food Safety Authority
FAO	Food and Agriculture Organization of the United Nations
FSS	Food Security and Sustainability
GAPDH	Glyceraldehyde 3-Phosphate Dehydrogenase
GC	Gas Chromatography
GC-MS	Gas Chromatography coupled with Mass Spectrometry
HEX1B	Hexamerin1B
ICP-MS	Inductively Coupled Plasma Mass Spectrometry
IgE	Immunoglobulin E
LC-MS/MS	Liquid Chromatography coupled with Tandem Mass Spectrometry
LC-Qtof-MS	Liquid Chromatography coupled with Quadrupole time-of-flight Mass Spectrometry
MS	Mass Spectrometry
PCB	Polychlorinated Biphenyls
SDS-PAGE	Sodium Dodecyl Sulfate-Polyacrylamide Gel Electrophoresis
US	United States

## Appendix A

**Table A1:** Most cited publications on edible insects in Mexico.

Title	Year	Journal	TC	TCy
Benefits and Challenges in the Incorporation of Insects in Food Products [177].	2021	Frontiers in Nutrition	54	13.50
An insight to fermented edible insects: A global perspective and prospective [94].	2020	Food Research International	40	8.00
Beyond Human Nutrition of Edible Insects: Health Benefits and Safety Aspects [195].	2022	Insects	40	13.33
Review: Insects-A Source of Safe and Sustainable Food?- “Jein” (Yes and No) [21].	2022	Frontiers in Sustainable Food Systems	11	3.67
Arthropods Accompanying Commercial Edible Grasshoppers, Sphenarium Charpentier1, from Outlets in Mexico City [152].	2020	Southwestern Entomologist	1	0.20
Nutritional Composition, Phenolic Compounds and Antioxidant Activity of Different Samples of Water Boatmen Eggs (Hemiptera: Corixidae) [9].	2023	Foods	0	0.00

TC = Total Citations; TCy = Total Citations per year.

**Table A2:** Market availability and pricing of edible insects in Oaxaca.

Location	Market Name	Product	Presentation	Cost (USD)	Quantity (g)
Tlacolula de Matamoros	Weekly Market *	Chapulines	Salty	2.5	120
Oaxaca City	20 de Noviembre	Chapulines	Spicy or Salty	2.7	120
Oaxaca City	Benito Juárez	Chapulines	Spicy or Salty	3	120
Oaxaca City	20 de Noviembre	Chicatanas	Salty	6	45
Oaxaca City	20 de Noviembre	Chicatanas	Salty	20	150
Oaxaca City	20 de Noviembre	Red Agave Worms	Live or dried	12.5	5
Villa de Zaachila	Weekly Market *	Chapulines	Spicy or Salty	2.65	120
Ocotlán de Morelos	Weekly Market *	Chapulines	Spicy or Salty	2.6	120
Asunción Nochixtlán	Weekly Market *	Chapulines	Spicy or Salty	2.75	120

* Weekly markets operate on designated days in different towns and villages, providing a diverse selection of local goods, including chapulines.
